# Identification of anti-schistosomal, anthelmintic and anti-parasitic compounds curated and text-mined from the scientific literature

**DOI:** 10.12688/wellcomeopenres.17987.1

**Published:** 2022-07-19

**Authors:** Avril Coghlan, Gilda Padalino, Noel M. O'Boyle, Karl F. Hoffmann, Matthew Berriman

**Affiliations:** 1 Wellcome Sanger Institute, Wellcome Genome Campus, Hinxton, Cambridge, CB10 1SA, UK; 2 The Institute of Biological, Environmental and Rural Sciences (IBERS), Aberystwyth University, Aberystwyth, Wales, SY23 3DA, UK; 3 School of Pharmacy and Pharmaceutical Sciences, Cardiff University, Redwood Building, King Edward VII Avenue, Cardiff, CF10 3NB, UK; 4 NextMove Software Ltd., Cambridge Science Park, Milton Rd., Cambridge, CB4 0WG, UK; 5 Sosei Heptares, Steinmetz Building, Granta Park, Great Abington, Cambridge, CB21 6DG, UK; 6 Wellcome Centre for Integrative Parasitology, Institute of Infection, Immunity and Inflammation, University of Glasgow, 120 University Place, Glasgow, G12 8TA, UK

**Keywords:** anthelmintic, drug, nematodes, flatworms, parasitic worms, compound, screen, anti-parasitic

## Abstract

More than a billion people are infected with parasitic worms, including nematodes, such as hookworms, and flatworms, such as blood flukes. Few drugs are available to treat worm infections, but high-throughput screening approaches hold promise to identify novel drug candidates. One problem for researchers who find an interesting ‘hit’ from a high-throughput screen is to identify whether that compound, or a similar compound has previously been published as having anthelmintic or anti-parasitic activity. Here, we present (i) data sets of 2,828 anthelmintic compounds, and 1,269 specific anti-schistosomal compounds, manually curated from scientific papers and books, and (ii) a data set of 24,335 potential anthelmintic and anti-parasitic compounds identified by text-mining PubMed abstracts. We provide their structures in simplified molecular-input line-entry system (SMILES) format so that researchers can easily compare ‘hits’ from their screens to these anthelmintic compounds and anti-parasitic compounds and find previous literature on them to support/halt their progression in drug discovery pipelines.

## Introduction

Parasitic nematodes and flatworms, commonly described as helminths, infect more than a billion people ( Diseases & Injuries, 2020). Helminth infections are usually long term, frequently resulting in chronic morbidity. The reliance on only a limited repertoire of anthelmintic drugs— *e.g.*, praziquantel, albendazole, mebendazole and ivermectin—and the vast numbers of doses administered per year, mean that there is an ever-present threat of drug resistance/drug insensitivity emerging. New affordable alternative drugs are constantly sought.

One problem for researchers who find an interesting ‘hit’ from a high-throughput screen is to find out whether that compound, or a similar compound has previously been published as having anthelmintic or anti-parasitic activity. This can be challenging because (i) compounds often have multiple common names, so searching for all papers that mention a particular compound is difficult; (ii) many published screens do not present the chemical structures in an easy-to-parse format to enable structure-based searches.

Ideally a researcher needs to have a file of all published hits as well as their chemical structures stored in the standard format, *e.g.*, simplified molecular-input line-entry system (SMILES) format ( Fourches *et al.*, 2010) in a plain text file or Excel spreadsheet (rather than for example, a pdf file, from which it is often hard to extract text). The recent publication of a screen by ( Knox *et al.*, 2021) is an exemplary case; names and SMILES of their ‘hits’ are provided in an Excel spreadsheet, which is easy to parse for use in subsequent analyses. Such a file can be read into chemistry analysis software, such as DataWarrior ( Sander *et al.*, 2015), to perform a ‘Similarity Analysis’ ( *e.g.*, Supplementary Figure 1, which can be found as *Underlying data* ( Coghlan, 2022)) to investigate whether a new hit compound is similar to any published anthelmintic compounds.

We previously published a curated set of SMILES for 261 known anthelmintic drugs and compounds, gathered from scientific papers and books (found in Supplementary Table 21 in ( International Helminth Genomes, 2019)), but that set was far from complete because it did not include results of high-throughput screens. Here we present (i) much larger curated data sets of 1,269 anti-schistosomal compounds and 2,828 other anthelmintic compounds curated from scientific papers and books, and (ii) a set of 24,335 potential anthelmintic and anti-parasitic compounds identified by text-mining PubMed abstracts.

## Methods

### Anthelmintic compounds curated from scientific papers

We initially gathered a list of compounds with published activity against helminths, primarily free-living nematodes, such as *Caenorhabditis elegans* and parasitic relatives, such as hookworms, but also some parasitic flatworms ( *e.g.*, tapeworms), from 29 different publications from 1994–2021 (Supplementary Table 1A, which can be found as *Underlying data*). Where provided, SMILES representations from original publications were stored directly. However, where compound names ( *e.g.*, ‘chlorpromazine’) rather than SMILES were provided in the original publications, we searched for the compound name in ChEMBL (RRID:SCR_014042) ( Mendez *et al.*, 2019) or PubChem (RRID:SCR_004284) ( Kim *et al.*, 2016) and took the SMILES from one of those databases. If it was absent from those databases we drew the compound in the Marvin JS v. 18.21.0 compound sketcher (ChemAxon) on the ChEMBL website ( Hastings *et al.*, 2016), or in the Ketcher compound sketcher ( Karulin & Kozhevnikov, 2011) on the ChEBI (RRID:SCR_002088) website and saved as SMILES from Marvin/Ketcher.

In total, 2,828 compounds with anthelmintic activity were curated, including those that we previously curated in ( International Helminth Genomes, 2019) (Supplementary Table 2, found as *Underlying data*). To identify duplicates (where a compound was curated from multiple sources), we read SMILES representations into DataWarrior v5.5.0 ( Sander *et al.*, 2015), selected the ‘Structure of SMILES’ column, and ran ‘Merge equivalent rows’ to merge rows with the same chemical structure. This revealed that there were 2,587 unique compounds (although this number still included stereoisomers, or different salt forms of the same parent compound).

### Anti-schistosomal compounds curated from scientific papers and books

We also gathered a more focussed list of compounds with published activity against the blood fluke *Schistosoma mansoni* and/or other *Schistosoma* species. These were based on 47 different publications from 1980–2021 (Supplementary Table 1B, found as *Underlying data*). In total, 1,269 compounds with anti-schistosomal activity were curated (Supplementary Table 3, see *Underlying data*). Using DataWarrior v5.5.0 ( Sander *et al.*, 2015) to merge duplicated compounds (see above), we found 1,115 unique compounds. Supplementary Figure 1 (see *Underlying data*) shows a ‘Similarity Analysis’ of the 1,269 compounds produced using DataWarrior based on the Skelspheres descriptor ( Boss *et al.*, 2017), a vector of integers which represents the occurrence of substructures in a compound. There are 123 different chemical classes labelled, which contain 378 compounds. The remaining 737 of the 1,269 compounds were ‘singletons’, that is they are relatively distinct in structure from any other published anti-schistosomal compound. In this analysis, salt forms of the same parent compound, or stereoisomers, were considered equivalent.

Note that the 123 chemical classes defined by DataWarrior consist of highly similar compounds, so that some related compounds such as the dihydropyridine drugs felodipine and nifedipine were placed in separate chemical classes by DataWarrior. The analysis in DataWarrior revealed cases where several compounds published separately belonged to the same chemical class; for example, pirarubicin ( Padalino *et al.*, 2018) and idarubicin ( Cowan & Keiser, 2015) are similar anthracyclines (labelled ‘class 95’ in Supplementary Figure 1, which can be found as *Underlying data*). Therefore, a future researcher who finds another similar anthracycline as a ‘hit’ in a screen could, by comparing their hit to our data sets, realise that their hit is similar to these previously published ‘hits’ by ( Padalino *et al.*, 2018) and ( Cowan & Keiser, 2015). Since we have recorded the literature source for each curated compound in our data sets, the researcher could then examine those papers to gain more information on the assays and activities for those previous hits.

### Putative anthelmintic and anti-parasitic compounds identified by text-mining PubMed abstracts

In addition, we have created a set of 24,335 potential anthelmintic and anti-parasitic compounds, by text-mining PubMed (RRID:SCR_004846) abstracts using the chemistry text-mining software LeadMine v 3.15.1 (NextMove Software Ltd.) ( Lowe & Sayle, 2015) to identify compounds in abstracts that also mentioned terms related to parasitic worms or other parasites. Free open source software that could be used to do similar tasks are OSCAR ( Jessop *et al.*, 2011) and Opsin ( Lowe *et al.*, 2011). The names and SMILES of 24,335 chemical compounds/elements were identified in 116,180 PubMed abstracts in February 2022 using LeadMine. LeadMine identifies chemical and biological terms within text, is aware of synonyms for chemical names and other terms, and can convert chemical names to SMILES format or resolve biological terms to ontologies. The 24,335 chemical compounds (Supplementary Table 4, see *Underlying data*) were identified in 116,180 PubMed abstracts that contained one or more terms relating to parasitic worms and anthelmintic/anti-parasitic compounds, such as ‘Schistosoma’, ‘nematode’, ‘anthelmintic’, ‘antiparasitic’, ‘malaria’, *etc.* (A full list of search terms can be found as *Underlying data* in Supplementary Table 5). Using DataWarrior v5.5.0 ( Sander *et al.*, 2015) to merge duplicated compounds (see above), we found 22,000 unique compounds.

The 24,335 compounds that LeadMine found in these PubMed abstracts were found by text-mining, rather than manual curation, so are potential (rather than experimentally confirmed) anthelmintic and anti-parasitic compounds. Indeed, some of the 24,335 compounds are obviously not anthelmintic, *e.g.*, water. However, even though this set contained some false positives, it likely contains many true anthelmintic and anti-parasitic compounds and has the added advantage that each compound is linked to one or more PubMed abstracts that mention it in conjunction with parasitic worms or other parasites (Supplementary Table 6, see *Underlying data*). Therefore, if a researcher finds a ‘hit’ in a screen for novel anthelmintic compounds and compares that hit to this set of 24,335 compounds ( *e.g.*, using DataWarrior), they may find compounds similar to their hit mentioned in PubMed abstracts that also mention parasites. Those papers can then be explored in more depth to understand the nature of the similar compounds and the experiments reported on them.

This approach was used in ( Wang *et al.*, 2020), in which we found simvastatin to be a ‘hit’ compound from a screen for anti-schistosomal compounds. Using DataWarrior to compare the SMILES of simvastatin to those of the set of 24,335 compounds text mined by LeadMine from PubMed abstracts, we found several papers on the anthelmintic and anti-parasitic activity of simvastatin. As LeadMine is aware of chemical synonyms, it identified PubMed abstracts that used synonyms of ‘simvastatin’, such as ‘mevinolin’ ( *e.g.*, ( Chen *et al.*, 1991)). In addition, since LeadMine provides the SMILES of compounds, by doing a ‘Similarity Analysis’ in DataWarrior, we were able to identify structurally similar compounds, *i.e.*, lovastatin, pravastatin, mevastatin, mevinolinic acid, and compactin, that are mentioned in PubMed abstracts related to parasites ( *e.g.*, ( Araujo *et al.*, 2002) and ( Haughan *et al.*, 1992) on the effects of lovastatin on *S. mansoni* and *Leishmania*, respectively).

## Dataset validation

The following are some caveats regarding the curated data sets of anthelmintic and anti-schistosomal compounds:

(i) **Duplicate compounds:** as mentioned above, each data set ( *e.g.*, the curated anthelmintic compounds) includes some duplicates due to curating the same compound from different sources. Even after merging compounds with identical SMILES, there are still some alternative salt forms of the same parent compound, or stereoisomers. There are also some of the same compounds present in both the anthelmintic set and the anti-schistosomal set.
(ii) **Sources of SMILES:** In some cases, the source publications included the SMILES for the compounds, in which case we took those SMILES. However, in many cases, just a name for the compound was given ( *e.g.*, ‘praziquantel’). In this case, we searched for the compound name in PubChem or ChEMBL and took the SMILES from PubChem/ChEMBL (see ‘Note on SMILES’ column in Supplementary Tables 2 and 3, found in *Underlying data*). However, if a vendor identifier for the compound was given in the source paper, we did not take the time to check whether the stereochemistry or salt form from PubChem/ChEMBL matched that given on the vendor’s website.
(iii) **Coverage of anthelmintic compounds:** The sets of anthelmintic and anti-schistosomal compounds are not a comprehensive set of all known anthelmintic/anti-schistosomal compounds, but even so we hope they will be a useful starting point for analyses. We have mostly focussed on large screens and therefore have missed many papers in the medicinal chemistry literature that focussed on particular chemical classes. In addition, in some cases a book or review paper mentioned the name of a compound, but we could not find its structure in PubChem or ChEMBL, so did not include it. Lastly, if several very similar active compounds were listed in a source paper, we just took one or a few of the most active compounds, as representatives of that compound class.
(iv) **Activity information:** These data sets were not intended to be a detailed record of activity information, which is for example included in ChEMBL ( Mendez *et al.*, 2019). We did not record whether the activity information came from *in vitro* data, *in vivo* experiments, clinical trials, or use as an approved drug; nor which worm species activity was observed for, at what concentration of compound, and what was the phenotype seen.
(v) **Natural extracts:** In some cases, a source paper listed several possible active constituents of a natural extract ( *e.g.*, a plant extract). In this case, we included all of these possible active constituents, with the idea that later researchers would be interested if they find an active compound similar to any of these.

Despite these caveats, we hope that, if in future a scientist finds a ‘hit’ compound, and if we had curated the same or a very similar compound, they could then re-examine the source papers from which we curated that compound, to glean extra information on stereochemistry, salt forms, activity, and possibly natural extracts. In addition, future researchers may wish to extend our data sets of curated compounds to include future screens and publish these to keep the data sets up to date.

Regarding our data set of compounds text-mined from PubMed abstracts that mention parasites, the main caveat is that the compounds are potential anthelmintic/anti-parasitic compounds. That is, some of the compounds could be ‘false positives’, for example, where a PubMed abstract about parasites mentions chemicals that are not anthelmintic/anti-parasitic, such as water or components of media. However, as for the curated anthelmintic/anti-schistosomal compounds, we hope that despite this, it could be useful for a future scientist to compare a ‘hit’ compound to these text-mined compounds, and if the same or a similar compound was text-mined from PubMed abstracts, to look at those abstracts and the associated papers to determine whether those papers do in fact record anthelmintic/anti-parasitic activity for the ‘hit’ compound or a similar compound.

Researchers who have identified ‘hits’ in high-throughput screens for anthelmintic/anti-parasitic activity often expend considerable time and effort in reading the literature to check whether the same compounds or similar compounds have been previously published as having activity. Since different researchers carry out their own manual checks, there is considerable duplication of effort. Here, we present data sets of manually curated anthelmintic compounds, as well as potential anthelmintic/anti-parasitic compounds that we text-mined from PubMed abstracts. While we originally created these data sets to investigate ‘hits’ from our own high-throughput screens against schistosomes and whipworms, we hope that this information will be useful to others who have carried out screens, and indeed lower the barrier for others to take on screens. By making this data ‘open’, we invite the community to keep it up to date by adding more curated ‘hits’ to it in future, ideally in the standard SMILES format so that the data is easy to parse and analyse. We welcome those who would like to extend the data set to contact us.

## Data availability

### Underlying data

Open Science Framework: Coghlan, Padalino et al_WOR. https://doi.org/10.17605/OSF.IO/JQP7N ( Coghlan, 2022).

This project contains the following underlying data:

- SupplementaryFig1_29jul2021alc.pptx (Supplementary Figure 1 shows a ‘Similarity Analysis’ of the 1,269 curated anti-schistosomal compounds produced using DataWarrior based on the Skelspheres descriptor, with chemical classes labelled. The text and borders around some of the chemical classes are in red or blue so that they can be distinguished from nearby classes)
- SuppTable1_17jun2022alc.xlsx (Supplementary Table 1 contains the literature sources of the curated anti-schistosomal and anthelmintic compounds)
- SuppTable2_17jun2022alc.xlsx (Supplementary Table 2 contains the SMILES of 2828 curated compounds with anthelmintic activity)
- SuppTable3_17jun2022alc.xlsx (Supplementary Table 3 contains the SMILES of 1269 curated compounds with anti-schistosomal activity)
- SuppTable4_17jun2022alc.xlsx (Supplementary Table 4 contains the list of compounds text mined by LeadMine from PubMed abstracts, and their SMILES)
- SuppTable5_17jun2022alc.xlsx (Supplementary Table 5 contains the list of words relating to helminths and parasites, which were searched for in PubMed abstracts)
- SuppTable6_17jun2022alc.xlsx (Supplementary Table 6 contains the compounds identified by LeadMine and the PubMed identifiers of the 116,180 PubMed abstracts in which they were found)

Data are available under the terms of the Creative Commons Zero "No rights reserved" data waiver (CC0 1.0 Public domain dedication).
