# Supplementary material for: Identification of anti-schistosomal, anthelmintic and anti-parasitic compounds curated and text-mined from the scientific literature
Source: Wellcome Open Res. 2022 Jul 19;7:193. [Version 1] doi: 10.12688/wellcomeopenres.17987.1 (PMC9363976; doi:10.12688/wellcomeopenres.17987.1)
Supplement: Peer review history

# Peer review: Reviewer response for version 1

**DOI:** 10.21956/wellcomeopenres.19944.r51603

**Published:** 2022-08-09

**Authors:** Josué de Moraes

**Affiliations:** 1 Research Center for Neglected Diseases, Guarulhos University, Guarulhos, Brazil

The authors describe a data set of anthelmintic compounds obtained from scientific articles and books. The topic is timely and interesting in the field of anthelmintic drug discovery. I have minor comments on the manuscript:

1. Authors have shown a database, but it is not clear whether it is possible to edit or add information for articles that will be published.
2. Regarding articles with several possible active constituents from natural products, did the authors consider compounds that were not tested?
3. I think the work would benefit from including some biological information (e.g. EC50).
4. Table should be revised. For example, anthelmintic compounds appear in Tables 1 and 2, while antischistosomal compounds appear in Tables 1 and 3. Note that an “antischistosomal” is an “anthelmintic”.

Are sufficient details of methods and materials provided to allow replication by others?

Yes

Is the rationale for creating the dataset(s) clearly described?

Yes

Are the datasets clearly presented in a useable and accessible format?

Yes

Are the protocols appropriate and is the work technically sound?

Yes

Reviewer Expertise:

Antiparasitic drug discovery

I confirm that I have read this submission and believe that I have an appropriate level of expertise to confirm that it is of an acceptable scientific standard.

# Peer review: Reviewer response for version 1

**DOI:** 10.21956/wellcomeopenres.19944.r51601

**Published:** 2022-08-08

**Authors:** Timothy Geary

**Affiliations:** 1 Institute of Parasitology, McGill University, Sainte-Anne-de-Bellevue, QC, Canada

The authors update a previous compendium and provide a highly useful dataset for scientists engaged in anthelmintic R&D. The manuscript is straightforward and clear. The addition of the SMILES system is a significant advance. There are no significant concerns with the manuscript, although 4 minor points are worth consideration by the authors:

1. Page 4: *"However, even though this set contained some false positives, it likely contains many true anthelmintic and anti-parasitic compounds...".* For accuracy, the authors should describe in more detail how they judged this to be "likely".
2. Page 4: *"The sets of anthelmintic and anti-schistosomal compounds are not a comprehensive set of all known anthelmintic/anti-schistosomal compounds.....".* Please comment on how this lack of completeness may limit the utility of the dataset.
3. *"In addition, future researchers may wish to extend our data sets of curated compounds to include future screens and publish these to keep the data sets up to date.".* Can the database be updated by other researchers? Can new compounds be added easily?
4. "SuppTable2_17jun2022alc.xlsx (Supplementary Table 2 contains the SMILES of 2828 curated compounds with anthelmintic activity)". Please note that 'anthelmintic' includes compounds with activity against nematodes, trematodes and cestodes; this table refers to compounds active against nematodes.

Are sufficient details of methods and materials provided to allow replication by others?

Yes

Is the rationale for creating the dataset(s) clearly described?

Yes

Are the datasets clearly presented in a useable and accessible format?

Yes

Are the protocols appropriate and is the work technically sound?

Yes

Reviewer Expertise:

Antiparasitic drug discovery and development; mechanisms of action and resistance to these drugs

I confirm that I have read this submission and believe that I have an appropriate level of expertise to confirm that it is of an acceptable scientific standard.
